# Non-Cardiac Surgery After Left Ventricular Assist Device Implantation: Perioperative Management and Outcomes from a Single Center

**DOI:** 10.3390/jcdd13100484

**Published:** 2026-09-26

**Authors:** Mustafa Akdi, Bekir Boğaçhan Akkaya, Abdulkadir Yılmaz, Sinan Sabit Kocabeyoğlu, Ümit Kervan

**Affiliations:** Department of Cardiovascular Surgery, Yuksek Ihtisas Heart Center, Ankara City Hospital, University of Health Sciences, Cankaya, 06800 Ankara, Türkiye; bullchan@gmail.com (B.B.A.); akykdrylmz@gmail.com (A.Y.); s4126k@yahoo.com.tr (S.S.K.); drukervan@yahoo.com (Ü.K.)

**Keywords:** left ventricular assist device, non-cardiac surgery, anticoagulation management, mortality

## Abstract

As survival after left ventricular assist device (LVAD) implantation improves, an increasing number of patients require non-cardiac surgery (NCS) during follow-up. This study aimed to evaluate the clinical characteristics and perioperative management of LVAD recipients undergoing NCS and to compare outcomes between elective and emergent procedures. Among 434 adults who underwent LVAD implantation between 2014 and 2026, 42 subsequently underwent NCS and were classified into elective (n = 26) and emergent (n = 16) groups. Overall, 9.68% of LVAD recipients required NCS, of which 61.9% were elective. Preoperative urea, creatinine, and international normalized ratio (INR) levels were significantly higher in the emergent group. Thirty-day mortality (31.2% vs. 3.8%; *p* = 0.023) and 1-year mortality (68.8% vs. 30.8%; *p* = 0.026) were significantly higher after emergent surgery. Kaplan–Meier analysis demonstrated significantly lower survival in the emergent group (log-rank *p* = 0.018). On univariable Cox regression analysis, emergent surgery was associated with a 2.87-fold higher mortality risk (HR, 2.87; 95% CI, 1.15–7.15; *p* = 0.024). NCS after LVAD implantation can be performed safely with appropriate patient selection, anticoagulation management, and multidisciplinary care; however, emergent surgery is associated with a less favorable preoperative clinical profile, limited opportunity for anticoagulation optimization, and substantially higher mortality.

## 1. Introduction

Heart failure is a major public health problem affecting more than 64 million people worldwide, associated with high mortality and an annual global economic burden exceeding USD 284 billion. In recent years, the increasing prevalence and poor prognosis of heart failure have accelerated research on left ventricular assist devices (LVADs), with the aim of improving survival and quality of life in patients with advanced heart failure [1,2].

Two landmark studies, the REMATCH and INTrEPID trials, demonstrated a survival benefit with LVAD support compared with optimal medical therapy in patients with advanced heart failure [3,4]. Advances in device technology, improved durability and performance, and growing clinical experience have contributed to reductions in early mortality. Consequently, LVADs are now increasingly used not only as a bridge to heart transplantation but also as destination therapy [5].

Improved survival among patients with LVADs has been accompanied by a parallel increase in the likelihood of developing non-cardiac comorbidities and conditions requiring surgical intervention. Surgical management in LVAD recipients is inherently complex because of the need for continuous anticoagulation, altered hemodynamics, and the risk of device-related complications. Furthermore, limited experience with LVAD physiology among non-cardiac surgical teams, together with the challenges associated with postoperative care, necessitates a multidisciplinary approach. These factors raise concerns regarding increased perioperative morbidity and mortality and may require modifications to standard surgical practices [6].

The available literature on non-cardiac surgery (NCS) in patients with LVADs remains limited and heterogeneous. Previous studies have shown that elective surgical procedures performed within a multidisciplinary care framework do not significantly affect long-term mortality, whereas emergent procedures are associated with increased early and late mortality. However, substantial differences exist across studies in terms of surgical indications, timing of surgery, anticoagulation strategies, and perioperative management practices [7,8,9].

The aim of this study is to characterize the clinical conditions requiring NCS during follow-up after LVAD implantation, determine their frequency and etiological distribution, and describe perioperative management strategies and institutional experience, thereby contributing to the identification of appropriate management approaches for this challenging patient population.

## 2. Materials and Methods

### 2.1. Study Design and Patient Population

This retrospective, single-center, observational cohort study was conducted after approval from the Institutional Ethics Committee. Since the initiation of our institutional LVAD program in 2014, all consecutive adult patients (≥18 years) who underwent durable LVAD implantation with a HeartWare HVAD (HeartWare, Framingham, MA, USA), HeartMate II (St. Jude Medical, St. Paul, MN, USA), or HeartMate 3 (St. Jude Medical, St. Paul, MN, USA) between 2014 and 2026 were retrospectively screened. Patients undergoing NCS after discharge following LVAD hospitalization were identified from our prospectively maintained electronic LVAD database. For patients with multiple NCS procedures, only the first was included. Patients were excluded if they underwent NCS during the initial LVAD hospitalization, underwent heart transplantation before NCS, had transitioned to biventricular assist device (BiVAD) support before NCS, or had insufficient clinical or follow-up data. LVAD-related procedures (e.g., driveline procedures or pump exchange) and diagnostic or nonsurgical procedures (e.g., cardiac catheterization or endoscopy) were not considered NCS. After application of these criteria, 42 patients were included in the study.

Surgical urgency was classified as elective and emergent. The emergent group encompassed all nonelective procedures requiring surgery within 24 h of presentation because of an acute or potentially life-threatening condition, including procedures requiring immediate intervention. Elective surgery was defined as a planned procedure for a non-urgent condition, allowing scheduled preoperative evaluation and optimization of anticoagulation and LVAD-related management (Figure 1).

Following discharge, LVAD follow-up was individualized according to clinical stability. Stable patients were evaluated at 2 weeks and 1 month, monthly until 6 months, and every 3 months thereafter, irrespective of subsequent NCS urgency. A dedicated 24/7 LVAD telephone line was available, and symptomatic patients could present directly to our cardiovascular surgery clinic without an appointment for prompt evaluation or be assessed by the on-call surgeon outside working hours. Patients living far from our center were referred to the nearest appropriately equipped hospital, when necessary, followed by physician-to-physician consultation and transfer to our center if indicated.

### 2.2. Data Collection

Demographic, perioperative, clinical, and laboratory data were retrospectively collected from the patients’ medical records.

- Demographic variables: Age, sex, body mass index (BMI), comorbidities (diabetes mellitus, peripheral arterial disease, atrial fibrillation, and history of stroke), presence of an implantable cardioverter-defibrillator (ICD) or permanent pacemaker, etiology of heart failure, type of LVAD (HeartWare HVAD, HeartMate II, or HeartMate 3), and indication for LVAD implantation.
- Variables related to NCS: Surgical indication, surgical specialty performing the procedure, time from LVAD implantation to NCS, center where the surgery was performed (whether the procedure was performed at a center other than the tertiary referral center where the LVAD was implanted), timing of surgery (elective or emergent), and anesthetic technique.
- Laboratory parameters: Urea, creatinine, estimated glomerular filtration rate (eGFR), alanine aminotransferase (ALT), aspartate aminotransferase (AST), lactate dehydrogenase (LDH), activated partial thromboplastin time (aPTT), international normalized ratio (INR), and platelet count, measured preoperatively and at 24 h postoperatively.
- Postoperative outcomes: Requirement for blood transfusion, length of hospital stay, 30-day mortality, and 1-year mortality.

The primary endpoint was 30-day mortality following non-cardiac surgery. Secondary endpoints included postoperative intensive care unit and hospital length of stay, and 1-year mortality.

### 2.3. Anticoagulation and Anesthesia Protocols and Perioperative LVAD Management

All patients scheduled for surgery were receiving warfarin sodium and acetylsalicylic acid (100 mg). In patients scheduled for elective surgery, combined therapy with warfarin sodium and acetylsalicylic acid was discontinued once the decision to proceed with surgery was made. Anticoagulation management was tailored according to the type and extent of the planned surgical procedure and the target INR determined by the surgical team. When the INR decreased below 2.0, bridging anticoagulation with enoxaparin was initiated at a dose of 100 IU/kg twice daily. Surgery was generally performed once the INR had decreased to <1.5.

Although the timing of postoperative antithrombotic therapy was individualized according to the type of surgical procedure and the patient’s bleeding risk, warfarin sodium and acetylsalicylic acid were restarted within the first 24 h after surgery in most patients once adequate surgical hemostasis had been achieved. Enoxaparin was administered as a single dose of 100 IU/kg on the evening of surgery and was subsequently continued at the same dose twice daily until the INR exceeded 2.0.

Following surgery, the warfarin sodium dose was adjusted to maintain a target INR of 2.0–3.0 in patients supported with the HeartWare HVAD or HeartMate II and 2.0–2.5 in those supported with the HeartMate 3. In HeartMate 3 recipients considered to be at high risk of bleeding, a lower target INR range of 1.8–2.3 was used.

In patients undergoing emergent surgery, reversal of anticoagulation was performed when clinically appropriate using agents such as fresh frozen plasma (FFP) and prothrombin complex concentrate (PCC), with a target INR of <2.0. In patients with thrombocytopenia or suspected platelet dysfunction, platelet replacement therapy with apheresis platelet concentrates was administered.

Before NCS, all patients underwent a comprehensive preoperative evaluation, including laboratory testing, assessment of LVAD parameters, and electrocardiographic evaluation for the detection of ventricular arrhythmias. Transthoracic echocardiography (TTE) was performed to assess right ventricular function, interventricular septal position, and preload status. Based on the echocardiographic findings, LVAD settings were optimized when clinically indicated.

For surgical procedures performed at our tertiary care center, an anesthesiologist experienced in LVAD physiology and a cardiovascular surgeon experienced in advanced heart failure surgery were present throughout the operation. The team was responsible for continuous monitoring of LVAD function and the patient’s hemodynamic parameters, with optimization as needed. During surgery, the LVAD controller and battery system were positioned to allow uninterrupted monitoring of device parameters and immediate adjustment by the heart failure surgeon when required. In patients with an ICD or permanent pacemaker, the device was reprogrammed or temporarily deactivated using a magnet after induction of anesthesia to prevent inappropriate device therapies related to electrocautery. All patients underwent invasive arterial blood pressure monitoring via an arterial catheter, as well as central venous catheterization and continuous electrocardiographic monitoring. External defibrillation pads were also placed to enable prompt treatment of perioperative arrhythmias.

Because LVAD flow is highly preload-dependent and sensitive to afterload, pump flow and pulsatility index were continuously monitored by the cardiovascular surgeon throughout the procedure. When necessary, pump speed (RPM) was adjusted to maintain optimal LVAD function and hemodynamic stability. In the early postoperative period, ICD function was reactivated when appropriate. TTE was repeated to reassess right ventricular function, interventricular septal position, and preload status, and LVAD settings were readjusted as needed based on the echocardiographic findings.

The majority of patients underwent general anesthesia with endotracheal intubation.

### 2.4. Statistical Analysis

Statistical analyses were performed using SPSS Statistics (version 22.0; IBM Corp., Armonk, NY, USA). Patients were divided into two groups according to the timing of surgery: elective and emergent. The normality of continuous variables was assessed using the Shapiro–Wilk test. Normally distributed variables were presented as mean ± standard deviation (SD) and compared between groups using the independent-samples *t*-test, whereas non-normally distributed variables were presented as median with interquartile range (IQR; 25th–75th percentile) and compared using the Mann–Whitney U test. For normally distributed variables, homogeneity of variance was assessed using Levene’s test. When Levene’s test was non-significant, the *t*-test result assuming equal variances was reported; when it was significant, the Welch–Satterthwaite-corrected result (equal variances not assumed) was reported.

Categorical variables were presented as frequencies and percentages [n (%)] and compared using contingency table analyses. Fisher’s exact test was used for 2 × 2 tables. For contingency tables larger than 2 × 2 in which more than 20% of cells had expected counts < 5, the Fisher–Freeman–Halton exact test was performed using the Exact Tests option rather than the asymptotic chi-square test, given the small overall sample size (n = 42).

Follow-up was calculated from the index NCS to death or the last confirmed follow-up date, with surviving patients censored at their last confirmed follow-up. Median follow-up duration was estimated using the reverse Kaplan–Meier method. Overall survival was estimated using the Kaplan–Meier method and compared between groups using the log-rank (Mantel–Cox) test. The association between surgical urgency (emergent vs. elective surgery) and all-cause mortality was evaluated using univariable Cox proportional hazards regression analysis. The effect estimate was reported as a hazard ratio (HR) with a 95% confidence interval (CI), with elective surgery serving as the reference category. A two-sided *p*-value < 0.05 was considered statistically significant for all analyses.

## 3. Results

Among 434 adult patients who underwent HeartWare HVAD, HeartMate II, or HeartMate 3 implantation at our center between 2014 and 2026, 42 (9.68%) required NCS during follow-up. Of these procedures, 61.9% were performed electively and 38.1% on an emergent basis.

### 3.1. Demographic Characteristics of Patients

Among the 42 LVAD recipients who underwent NCS, no statistically significant differences were observed between the elective and emergent surgery groups in terms of age, sex, BMI, or comorbidities, including diabetes mellitus, peripheral arterial disease (PAD), pre-existing atrial fibrillation (AF), presence of a permanent pacemaker or ICD, and history of stroke. Similarly, the groups did not differ significantly with respect to heart failure etiology, indication for LVAD implantation, or LVAD type (*p* = 0.842, *p* = 0.281, and *p* = 0.120, respectively) (Table 1).

### 3.2. Characteristics of Non-Cardiac Surgical Procedures

Gastrointestinal surgery was the most frequently performed procedure in the elective surgery group, whereas neurosurgical procedures were the most common in the emergent surgery group. Peripheral vascular surgery was the second most frequently performed type of NCS in both groups (Table 2). Most procedures performed by the gastrointestinal surgery, orthopedic surgery, obstetrics and gynecology, thoracic surgery, and plastic and reconstructive surgery departments were elective, whereas the majority of neurosurgical procedures were performed under emergent conditions (Figure 2).

The mean interval between LVAD implantation and the first NCS was 752.2 ± 745.3 days, with a median of 601 days. The interval ranged from 5 to 2746 days, with an interquartile range (IQR) of 141.5–1058 days, indicating substantial variability in the timing of surgical intervention. Although the interval between LVAD implantation and NCS tended to be shorter in the elective surgery group, the difference did not reach statistical significance (*p* = 0.679).

Seven patients (43.8%) in the emergent surgery group and 11 patients (42.3%) in the elective surgery group underwent NCS within the first year after LVAD implantation. There was no statistically significant difference between the groups in the proportion of patients undergoing NCS within the first year.

No statistically significant association was observed between surgical urgency and the anesthetic technique used (*p* = 0.43). Similarly, there was no significant difference between the groups according to the center where NCS was performed (the tertiary care center where LVAD implantation was performed vs. another center; *p* = 1.00) (Table 3).

### 3.3. Preoperative and Postoperative Laboratory Findings

Preoperatively, urea and creatinine levels were significantly higher in the emergent surgery group than in the elective surgery group (urea: 54.9 ± 22.0 vs. 41.6 ± 14.8 mg/dL, *p* = 0.025; creatinine: 1.31 ± 0.64 vs. 0.96 ± 0.34 mg/dL, *p* = 0.028). Although eGFR was lower in the emergent surgery group, the difference did not reach statistical significance (*p* = 0.064). No significant differences were observed between the groups in preoperative liver enzyme levels (ALT and AST), LDH, aPTT, or platelet count. Preoperative INR was significantly higher in the emergent surgery group than in the elective surgery group.

At 24 h postoperatively, urea and creatinine levels were higher, and eGFR was lower in the emergent surgery group; however, none of these differences reached statistical significance (*p* = 0.282, *p* = 0.072, and *p* = 0.095, respectively). Similarly, no significant differences were observed between the groups in postoperative liver enzyme levels, LDH, aPTT, or platelet count (Table 4).

### 3.4. Postoperative Clinical Outcomes

There was no significant difference in the requirement for postoperative blood transfusion between LVAD recipients undergoing emergent and elective surgery (43.8% vs. 61.5%, *p* = 0.344). Although the length of hospital stay was longer in the emergent surgery group, the difference was not statistically significant. Thirty-day mortality was significantly higher in the emergent surgery group (31.2% vs. 3.8%, *p* = 0.023). Similarly, 1-year mortality was significantly higher in the emergent surgery group than in the elective surgery group (68.8% vs. 30.8%, *p* = 0.026) (Table 5).

Among the six patients who died within 30 days after NCS, one was in the elective group, and five were in the emergent group. The single death in the elective group occurred on postoperative day 18 due to right ventricular failure secondary to acute kidney injury. In the emergent group, deaths occurred between postoperative days 8 and 27 and were attributed to sepsis (n = 2), hemorrhagic or ischemic stroke (n = 2), and subarachnoid hemorrhage (n = 1) (Table 6).

The median follow-up duration after NCS, estimated using the reverse Kaplan–Meier method, was 949 days (31.2 months). Vital status was available for all 42 patients, with no patients lost to follow-up in either the elective or emergent group. Kaplan–Meier survival analysis demonstrated significantly lower survival in the emergent surgery group (log-rank *p* = 0.018). Univariable Cox proportional hazards regression analysis showed that emergent surgery was associated with an approximately 2.9-fold higher risk of mortality compared with elective surgery (HR, 2.87; 95% CI, 1.15–7.15; *p* = 0.024) (Figure 3).

## 4. Discussion

Recent advances in LVAD technology have substantially improved survival among LVAD recipients, resulting in a growing number of patients requiring NCS during long-term follow-up. Consequently, the perioperative management of these patients has become an increasingly relevant challenge in clinical practice [10]. The reported frequency of NCS among LVAD recipients varies considerably across studies, likely reflecting differences in patient population size and clinical characteristics, as well as the types of surgical procedures performed across centers [8,11,12]. In a large-scale study including 30,323 patients who underwent LVAD implantation, 10.2% subsequently required NCS, of which 73.5% were performed on an emergent basis [7]. In our cohort, 9.6% of LVAD recipients underwent NCS during follow-up, a rate comparable to that reported in previous studies, and the majority of procedures (61.9%) were performed electively.

LVAD recipients may undergo NCS across a wide range of surgical specialties, including general/abdominal surgery, neurosurgery, vascular surgery, orthopedics, thoracic surgery, head and neck surgery, genitourinary surgery, and plastic and reconstructive surgery [7,11,12,13]. General/abdominal surgery, peripheral vascular surgery, and orthopedic surgery are among the most frequently performed procedures, whereas studies evaluating emergent and elective procedures separately have identified neurosurgery and head and neck surgery as prominent specialties among emergent cases [7]. In our study, NCS procedures were analyzed according to surgical urgency as elective or emergent. Consistent with previous reports, elective procedures were most commonly gastrointestinal, vascular, and orthopedic surgeries, whereas neurosurgical procedures predominated in the emergent group. No significant differences were observed between the two groups in the proportion of patients undergoing NCS within the first year after LVAD implantation or in the interval from LVAD implantation to NCS. Furthermore, the comparable baseline demographic and clinical characteristics of the two groups reduced the potential influence of baseline differences when interpreting between-group outcomes.

At our center, the majority of NCS procedures were performed under general anesthesia. Although general anesthesia was the most frequently used technique in both groups, anesthetic approaches were more heterogeneous in the elective surgery group. Nevertheless, there was no significant difference in anesthetic techniques between the elective and emergent groups. Similarly, previous studies have reported that most surgical procedures in LVAD recipients are performed under general anesthesia [14]. Although regional anesthesia is not absolutely contraindicated in patients with LVADs, evidence supporting the use of spinal anesthesia in this population remains limited, and neuraxial anesthesia is rarely performed, accounting for less than 1% of cases [15]. In our cohort, spinal anesthesia was used in three patients (7%) undergoing elective surgery, with no spinal anesthesia-related complications observed.

Perioperative outcomes of emergent surgery in LVAD recipients depend on the timely assessment and optimal management of multiple factors, including the underlying pathology, the planned surgical procedure, and the patient’s bleeding and thrombotic risk profile. Preoperative anticoagulation status is an important determinant of perioperative outcomes. According to our institutional protocol, warfarin sodium and acetylsalicylic acid are discontinued in patients scheduled for surgery, while anticoagulation is maintained using a bridging strategy with low-molecular-weight heparin. Similarly, Beetz et al. used heparin bridging for at least 48 h following discontinuation of oral vitamin K antagonists [16]. Previous reports have indicated that active anticoagulation reversal strategies, such as vitamin K, PCC, or FFP, are not routinely used for elective surgical procedures [17]. In our study, preoperative INR was significantly higher in the emergent surgery group than in the elective group; however, there was no significant difference between the groups in the requirement for postoperative blood transfusion. In contrast, Morgan et al. reported that higher INR levels were associated with increased transfusion requirements and bleeding complications [11]. Arnaoutakis et al. also reported postoperative bleeding in two patients undergoing emergent surgery, which was attributed to the inability to adequately adjust anticoagulation before surgery [18]. Owing to the retrospective design of our study, data regarding the amounts of FFP, PCC and platelet concentrates administered during the postoperative period were incomplete. Therefore, these parameters could not be compared between the groups, and it remains unclear whether the absence of a difference in blood transfusion requirements was influenced by the administration of FFP, PCC, or platelet concentrates. This represents one of the limitations of our study. Although preoperative renal function was worse in the emergent surgery group, no significant difference in postoperative renal function was observed between the groups.

An important finding of our study was the marked adverse association between emergent surgery and mortality. Kaplan–Meier survival analysis demonstrated a clear separation between the elective and emergent surgery groups, with significantly lower survival in the emergent group during both the early and late postoperative periods. Furthermore, emergent surgery was associated with an approximately 2.87-fold higher risk of mortality. Beetz et al. reported a 30-day mortality rate of 47.1% among LVAD recipients undergoing emergent abdominal surgery, while demonstrating that elective procedures could be performed safely [16]. Similarly, Mathis et al. showed that emergent NCS was associated with significantly increased early mortality [19]. In a large study including 8118 patients, 30-day mortality was 4.3% after elective procedures and 10.2% after emergent procedures across various surgical specialties [20]. Briasoulis et al. further identified emergent surgery as an independent risk factor for mortality [7]. The higher mortality observed after emergent surgery may be attributable to several factors. A detailed examination of the causes and timing of death provides further insight into this association. Among the six patients who died within 30 days, one was in the elective group (day 18), whereas five were in the emergent group (days 8, 8, 8, 15, and 27). Notably, early deaths in the emergent group were predominantly associated with infectious, neurological, and vascular complications, which may reflect the greater baseline clinical acuity of patients requiring nonelective surgery. The higher mortality observed after emergent NCS may therefore be related to the combined effects of greater baseline clinical severity, impaired physiological reserve, as suggested by significantly higher preoperative urea, creatinine, and INR levels, and limited opportunity for preoperative anticoagulation optimization, rather than surgical urgency alone. The more favorable outcomes observed after elective surgery support the feasibility of performing NCS safely in LVAD recipients when appropriate patient selection, careful optimization of anticoagulation, and a multidisciplinary perioperative approach are implemented.

In our study, the center where NCS was performed (the tertiary care center where LVAD implantation was performed vs. another center) was not significantly associated with mortality. Nevertheless, the unique hemodynamic characteristics of LVAD recipients are well recognized to pose considerable challenges to perioperative management [21]. Therefore, despite the absence of a significant center-related difference in mortality in our cohort, the importance of performing major surgical procedures at experienced referral centers, whenever feasible, should not be overlooked. These findings should, however, be interpreted with caution given the limited sample size of our study.

## 5. Limitations

The relatively small sample size of this study limited the feasibility of subgroup analyses and multivariable statistical modeling, as well as the generalizability of the findings. Therefore, the association between emergent surgery and mortality was evaluated using univariable Cox proportional hazards regression analysis, and a multivariable analysis could not be performed. In addition, incomplete records regarding the amounts of fresh frozen plasma, prothrombin complex concentrate, and platelet concentrates administered during the postoperative period precluded a detailed assessment of the potential effects of these interventions on clinical outcomes.

## 6. Conclusions

This study provides a detailed real-world characterization of elective and emergent NCS in LVAD recipients, integrating the spectrum and timing of surgical procedures with perioperative anticoagulation management, intraoperative LVAD management, and clinical outcomes. Patients undergoing emergent NCS presented with a less favorable preoperative clinical profile and experienced substantially higher early and late mortality, reflecting the complex clinical context in which nonelective procedures are performed. These findings highlight the importance of considering surgical urgency together with the underlying clinical condition and perioperative management when assessing the risk of NCS in LVAD recipients. Larger multicenter studies are needed to determine whether standardized perioperative management strategies can improve outcomes in this high-risk population.

## Figures and Tables

**Figure 1:**
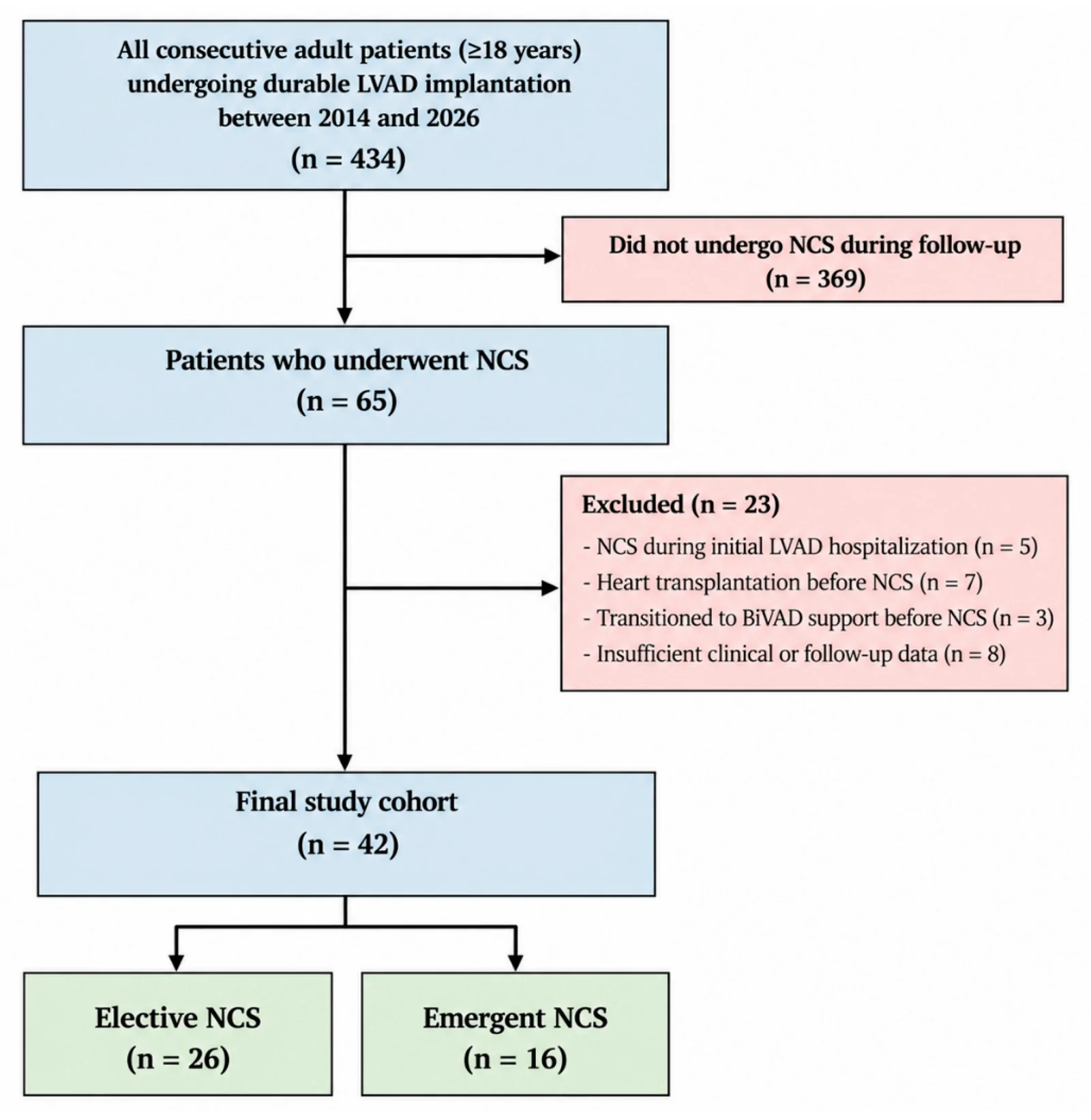
Flowchart of patient screening and inclusion in the study. LVAD: left ventricular assist device; NCS: non-cardiac surgery; BiVAD: biventricular assist device.

**Figure 2:**
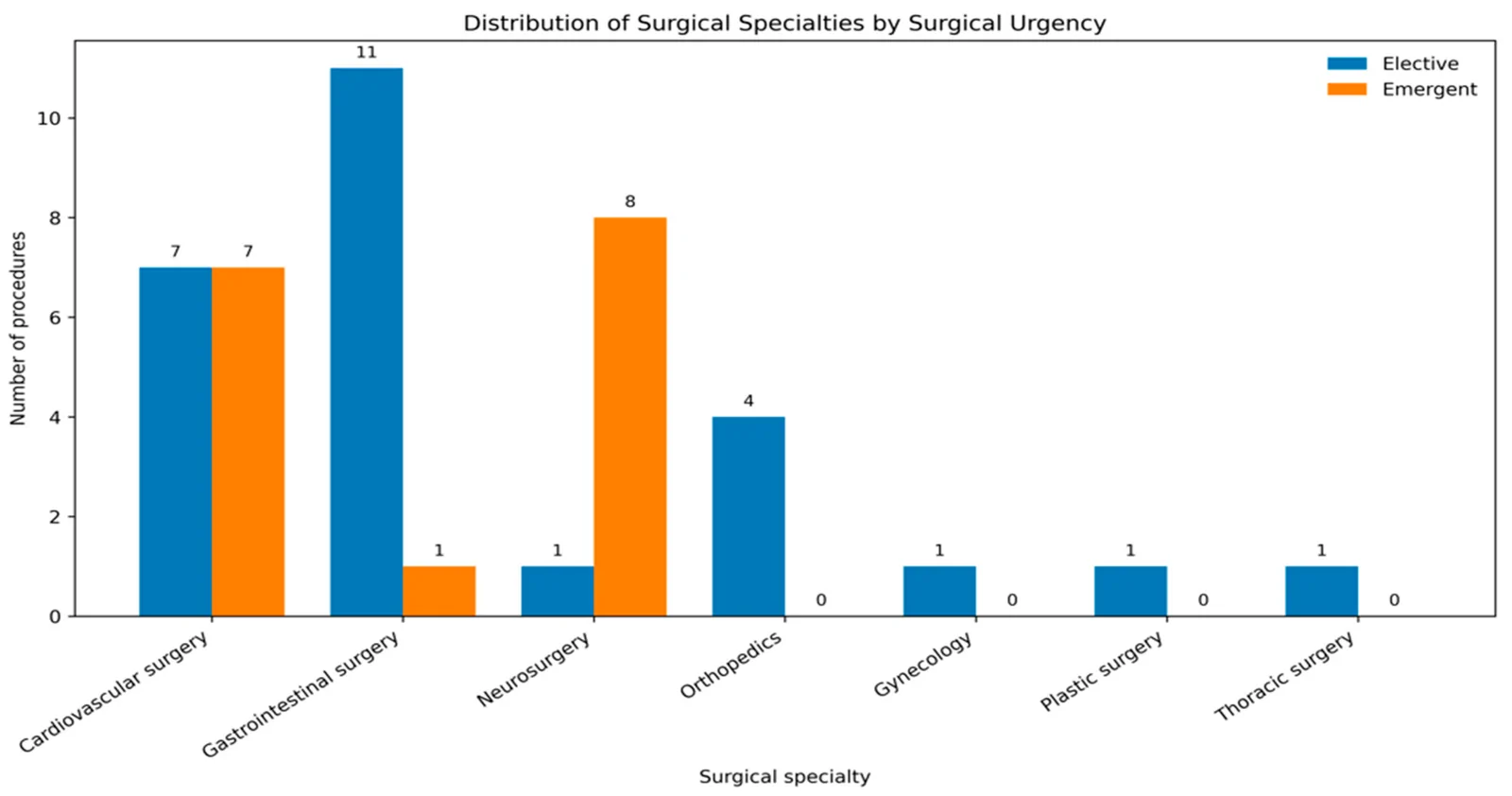
Distribution of surgical specialties according to surgical urgency in LVAD patients undergoing noncardiac surgery.

**Figure 3:**
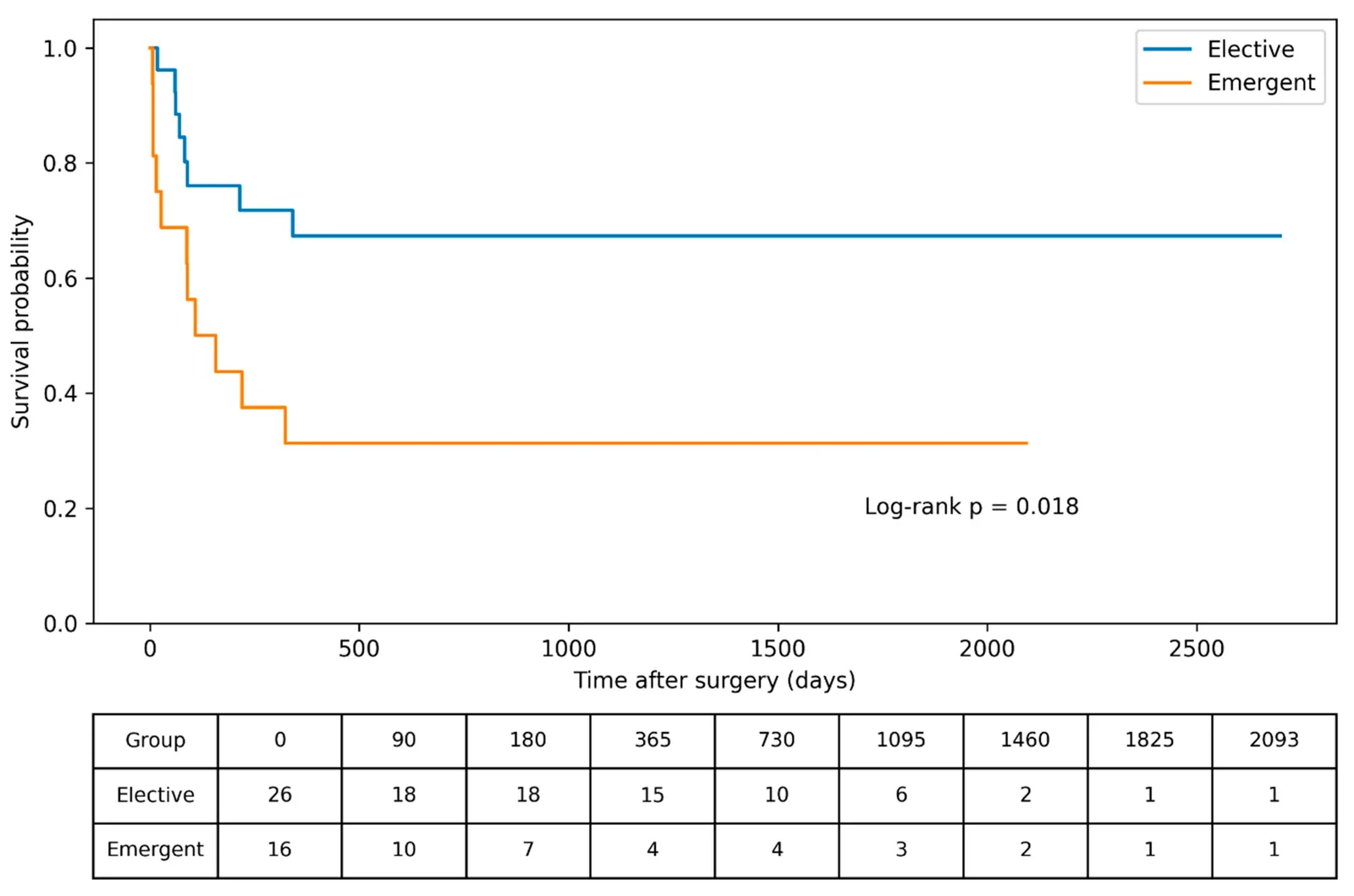
Kaplan–Meier survival curves demonstrating early divergence of the survival curves and significantly reduced survival in the emergent group. Numbers at risk at selected time points are shown below the plot.

**Table 1:** Baseline Characteristics of Patients Undergoing Elective vs. Emergent NCS After LVAD Implantation.

Variables	Elective (n = 26)	Emergent (n = 16)	*p* Value
Age, years (mean ± SD)	45.4 ± 10.5	45.9 ± 14.9	0.889
Sex, male, n (%)	20 (76.9)	14 (87.5)	0.688
BMI, kg/m^2^ (mean ± SD)	24.7 ± 4.0	24.6 ± 5.1	0.910
Diabetes mellitus, n (%)	6 (23.1)	5 (31.2)	0.720
PAD, n (%)	1 (3.8)	3 (18.8)	0.146
Preexisting AF, n (%)	3 (11.5)	2 (12.5)	1.000
Permanent pacemaker/ICD, n (%)	21 (80.8)	15 (93.8)	0.380
Stroke, n (%)	3 (11.5)	1 (6.2)	1.000
Heart failure etiology, n (%)			0.842
Dilated cardiomyopathy	16 (61.5)	9 (56.2)	
Ischemic cardiomyopathy	9 (34.6)	7 (43.8)	
Other	1 (3.8)	0 (0)	
LVAD indication, n (%)			0.281
Bridge to transplantation	21 (80.8)	10 (62.5)	
Destination therapy	5 (19.2)	6 (37.5)	
Device type, n (%)			0.120
HeartWare	7 (26.9)	6 (37.5)	
HeartMate II	0 (0)	2 (12.5)	
HeartMate 3	19 (73.1)	8 (50.0)	

SD: standard deviation; BMI: body mass index; PAD: peripheral artery disease; AF: atrial fibrillation; ICD: implantable cardioverter defibrillator; NCS: non-cardiac surgery; LVAD: left ventricular assist device.

**Table 2:** List of Non-cardiac Surgical Procedures in LVAD Patients.

Surgical Speciality	Surgical Urgency	NCS Procedure	n
Neurosurgery	elective	Subarachnoid hematoma evacuation	1
	emergent	Subdural hematoma evacuation	7
		Cranial decompression	1
Cardiovascular surgery	elective	Femoral embolectomy	1
		Femoral pseudoaneurysm repair	3
		Rectus sheath hematoma repair	1
		Aorto-bi-iliac graft	1
		Iliofemoral bypass and embolectomy	1
	emergent	Femoral embolectomy	7
Gastrointestinal surgery	elective	Colostomy	3
		Umbilical hernia repair	3
		Anal abscess drainage	1
		Anal fissure surgery	1
		Cholecystectomy	1
		Limberg flap	1
		Gluteal abscess drainage	1
	emergent	Splenic rupture surgery	1
Orthopedics	elective	Femur fracture surgery	1
		Hip fracture surgery	1
		Radius malunion repair	1
		Amputation	1
	emergent	-	
Thoracic surgery	elective	Upper lobectomy	1
	emergent	-	
Plastic surgery	elective	Rectus abdominis flap	1
	emergent	-	
Gynecology	elective	Bartholin abscess drainage	1
	emergent	-	

LVAD: left ventricular assist device; NCS: non-cardiac surgery.

**Table 3:** Procedural Characteristics of Non-cardiac Surgery According to Surgical Urgency.

Variables	Elective (n = 26)	Emergent (n = 16)	*p* Value
Time from LVAD implantation to surgery, days, median (IQR)	566 (183–1028)	613 (115–1059)	0.679
Surgery within the first year			1.000
Yes	11 (42.3)	7 (43.8)	
No	15 (57.7)	9 (56.2)	
Anesthesia type, n (%)			0.351
Local	5 (19.2)	5 (31.2)	
Spinal	3 (11.5)	0 (0)	
General	18 (69.2)	11 (68.8)	
Surgical center, n (%)			0.098
Non-tertiary	6 (23.1)	8 (50.0)	
Tertiary	20 (76.9)	8 (50.0)	

LVAD: left ventricular assist device; IQR: interquartile range.

**Table 4:** Comparison of Preoperative and Postoperative Laboratory Parameters Between Elective and Emergent Surgery Groups.

Variables		Elective (n = 26)	Emergent (n = 16)	*p* Value
Preoperative laboratory parameters				
Urea (mg/dL)	mean ± SD	41.6 ± 14.8	54.9 ± 22.0	**0.025**
Creatinine (mg/dL)	mean ± SD	0.96 ± 0.34	1.31 ± 0.64	**0.028**
eGFR (mL/min/1.73 m^2^)	mean ± SD	90.6 ± 26.0	74.2 ± 35.8	0.093
ALT (U/L)	median (IQR)	19.0 (12.5–28.3)	19.5 (13.8–35.0)	0.622
AST (U/L)	median (IQR)	18.5 (13.5–30.3)	24.0 (15.8–42.0)	0.414
LDH (U/L)	median (IQR)	235.5 (202.8–310.3)	257.0 (221.3–310.5)	0.688
aPTT (sec)	median (IQR)	37.1 (31.7–39.3)	37.3 (34.4–43.0)	0.233
INR	median (IQR)	1.80 (1.60–2.10)	2.45 (2.10–2.73)	**0.002**
Platelet (×10^3^/µL)	median (IQR)	225.5 (176.5–279.3)	180.5 (144.0–288.0)	0.174
Postoperative laboratory parameters				
Urea (mg/dL)	median (IQR)	46.0 (32.5–53.8)	51.5 (38.3–64.5)	0.282
Creatinine (mg/dL)	median (IQR)	0.94 (0.77–1.15)	1.15 (0.97–1.44)	0.072
eGFR (mL/min/1.73 m^2^)	mean ± SD	90.3 ± 26.8	73.7 ± 36.0	0.095
ALT (U/L)	median (IQR)	14.0 (9.3–27.5)	19.0 (13.8–29.0)	0.500
AST (U/L)	median (IQR)	19.0 (13.0–46.3)	25.5 (16.8–65.5)	0.218
LDH (U/L)	median (IQR)	250.0 (213.3–324.5)	292.0 (188.3–341.5)	0.806
aPTT (sec)	median (IQR)	36.9 (33.2–41.0)	33.3 (29.6–44.7)	0.430
Platelet (×10^3^/µL)	mean ± SD	245.9 ± 102.2	213.9 ± 94.5	0.318

eGFR: estimated glomerular filtration rate; ALT: alanine aminotransferase; AST: aspartate aminotransferase; LDH: lactate dehydrogenase; aPTT: activated partial thromboplastin time; INR: international normalized ratio; SD: standard deviation; IQR: interquartile range. Significant *p*-values are shown in bold (*p* < 0.05).

**Table 5:** Comparison of Clinical Outcomes of Elective vs. Emergent NCS.

Variables	Elective (n = 26)	Emergent (n = 16)	*p* Value
Blood transfusion requirement, n (%)	16 (61.5)	7 (43.8)	0.344
Hospital length of stay, days, median (IQR)	17.0 (11.3–25.8)	22.5 (8.0–33.0)	0.534
30-day mortality, n (%)	1 (3.8%)	5 (31.2%)	**0.023**
1-year mortality, n (%)	8 (30.8%)	11 (68.8%)	**0.026**

IQR: interquartile range; NCS: non-cardiac surgery. Significant *p*-values are shown in bold (*p* < 0.05).

**Table 6:** Causes and Timing of 30-Day Mortality Following NCS.

Surgical Urgency	NCS Procedure	Cause of Death	Time from NCS to Death (Days)
Elective	Femoral embolectomy	AKI, right ventricular failure	18
Emergent	Femoral embolectomy	Haemorrhagic stroke	15
	Femoral embolectomy	Sepsis	8
	Iliofemoral bypass and embolectomy	Sepsis	8
	Subdural hematoma evacuation	Subarachnoid hemorrhage	27
	Cranial decompression	Ischemic stroke	8

NCS: non-cardiac surgery; AKI: acute kidney injury.

## Data Availability

The datasets generated and/or analyzed during the current study are only available from the corresponding author on reasonable request due to institutional and ethical restrictions on data privacy.
